# Presence of Increased Mast Cells in Infants and Children with Volume and Variety Limited Intake

**DOI:** 10.3390/nu14020365

**Published:** 2022-01-15

**Authors:** Amy Issa, Jensen Edwards, Meenal Singh, Craig Friesen, Sarah Edwards

**Affiliations:** 1 Division of Gastroenterology, University of Texas Health Science Center at San Antonio, San Antonio, TX 78229, USA; issaa@uthscsa.edu; 2 School of Medicine, Texas Tech University Health Sciences Center El Paso, El Paso, TX 79905, USA; jensen.edwards@ttuhsc.edu; 3 Division of Gastroenterology, Children’s Mercy Kansas City, Kansas City, MO 64108, USA; msingh1@cmh.edu (M.S.); cfriesen@cmh.edu (C.F.); 4 School of Medicine, University of Missouri at Kansas City, Kansas City, MO 64108, USA

**Keywords:** feeding disorder, mast cells, pathology, etiology, eosinophils

## Abstract

Background: Reports indicate patients with feeding difficulties demonstrate signs of inflammation on biopsies, notably eosinophilia, but it is unknown whether mast cell density contributes to variety or volume limitation symptoms. The aim of our study was to evaluate eosinophil and mast cell density of EGD biopsies in pediatric patients with symptoms of decreased volume or variety of ingested foods. Methods: We conducted a single-center, retrospective chart review of EMRs for all new feeding clinic patients between 0 and 17 years of age. Patients were categorized by symptoms at the initial visit as well as eosinophil and mast cell densities in those with EGD biopsies. Ten patients were identified as controls. Results: We identified 30 patients each with volume and variety limitation. Antral mast cell density was increased in 32.1% of variety-limited patients, 37.5% of volume limited patients, and in no controls; Duodenal mast cell density was increased in 32.1% of variety-limited patients, 40.6% of volume-limited patients, and in no controls. Conclusions: In both variety- and volume-limited patients, antral and duodenal mast cell densities were increased. These associations warrant further investigation of the mechanism between mast cells and development of feeding difficulties, allowing more targeted pediatric therapies.

## 1. Introduction

Symptoms of feeding difficulties (FDs) can be challenging to ascertain in the pediatric population, especially among younger children and those who are non-verbal. FDs are present in 50% of children with normal development and 80% in children with developmental delays [1]. The etiology of FDs in children is often multifactorial, including medical, oral motor, and behavioral causes [2]. It is well documented that infants and children with feeding disorders have a higher incidence of gastrointestinal disorders with more recent literature highlighting specifically the increase in eosinophils in patients with feeding disorders [1,2,3,4,5]. GI pathology can significantly alter feeding patterns. Sdravou et al. noted that children with GI conditions can develop a restricted diet, practice selectivity, feeding refusal, and acquire general anxiety associated with feeding [3].

Some children may have difficulty articulating abdominal pain or nausea, which can manifest as issues that arise with feeding, such as decreased volume of intake and decreased variety of foods ingested. Mucosal inflammation, including the presence of mast cells and eosinophils, may contribute to these symptoms. Increased eosinophil and mast cell degranulation has been demonstrated in both children and adults with functional dyspepsia [6,7,8]. Mast cells have been evaluated in children with functional dyspepsia with nausea vs. patients with functional abdominal pain without nausea, and the number of duodenal mast cells was higher in those patients with functional dyspepsia with nausea [9]. Increased mast cell density is also associated with slower gastric emptying and increased gastric dysrhythmia in children with functional dyspepsia [10]. In patients with eosinophilic esophagitis, higher tryptase levels are associated with increased frequency of nausea [11]. A recent study in pediatric patients with rumination syndrome demonstrated increased eosinophil and mast cell densities in the gastric antrum [12]. Reports indicate a high proportion of patients with feeding difficulties who have undergone esophagogastroduodenoscopy (EGD), demonstrate some degree of inflammation on biopsies [4,13], with a subset including those with eosinophilia [14]. It is unknown whether mast cell density and eosinophilia contribute to symptoms of decreased volume or variety of foods ingested in these children.

Diagnosing the primary contributing factors of FDs should be determined urgently, due to risk of malnutrition, delayed neurologic development, and continued altered feeding patterns, which are difficult behaviors to change. The aim of our study was to evaluate mucosal biopsies obtained from EGDs for eosinophil and mast cell density in pediatric patients with feeding difficulties and symptoms of decreased volume or variety of ingested foods. 

## 2. Materials and Methods

We conducted a single-center, retrospective chart review of electronic medical records (EMRs) for all new patients between the ages of 0 and 17 years presenting to the Multidisciplinary Feeding Clinic at Children’s Mercy Hospital between 1 October 2014 and 31 December 2019. EMRs were reviewed for sex, age at the time of referral, clinic notes, symptoms, presence of a feeding tube, and endoscopic findings including histology. These data were entered into a secure, restricted database. The Children’s Mercy Kansas City Institutional Review Board approved the study.

All of the patients were further categorized by symptoms present at the initial clinic visit. The symptoms analyzed for this study were limited variety of foods and limited volume of food intake. Symptoms were obtained based on parent or patient report and are part of the standard questions asked at each Multidisciplinary Feeding Clinic visit. Limited variety was identified by a combination of notation of limited number of foods the child would eat, along with caregiver report of need for increased variety in the patient’s diet. Limited volume was identified by a combination of notation of small volume of intake or intolerance to larger volumes of intake, along with caregiver report of need for increased volume in the patient’s diet. 

For patients who had EGDs, previously obtained specimens from the lower esophagus, gastric antrum, and duodenum were evaluated by board-certified pediatric pathologists as part of routine clinical care. Patients did not have any histologic evidence of IBD, celiac disease, h.pylori and giardiasis. Patients were also negative for any recent infection. In addition, all antral and duodenal specimens were independently assessed by a single observer (M.S.) for eosinophil and mast cell densities. Each patient had two gastric and four duodenal biopsies. Eosinophil densities were assessed utilizing hematoxylin and eosin (H&E) stained slides. For mast cell assessment, slides underwent immunohistochemical (IHC) staining for tryptase employing anti-human mast cell tryptase (Dako; Clone AA1). To determine cell densities, sections were initially scanned at 10× objective magnification to determine subjective areas of greatest density. Then, five consecutive high-power fields (hpf) were assessed at 40× magnification to determine mean and peak densities. Only cells within the lamina propria were assessed. 

Patients who had EGDs were categorized by volume and variety limited subtypes, and patients who were volume- but not variety-limited, and a separate group who were variety- but not volume-limited, were identified for assessment. Ten patients were identified as controls from our pathology database who had previously had an EGD, who had a chief complaint of constipation without any other gastrointestinal symptoms. All controls had a minimum follow up period of 3 years post endoscopy demonstrating no pathology. 

All statistical analyses were performed using SPSS version 23 (SPSS, Inc.; Chicago, IL, USA). Categorical variables (e.g., presence of individual symptoms, presence of eosinophils, etc.) were compared using the Chi-Squared test or Fischer’s exact test, whichever was appropriate. Continuous variables, such as age, were compared using student’s *t*-test or ANOVA for multiple group comparisons. A *p*-value < 0.05 was considered significant. For all mast cell counts, pairwise comparisons were performed with Bonferroni corrections performed for multiple tests.

## 3. Results

**Figure 1:**
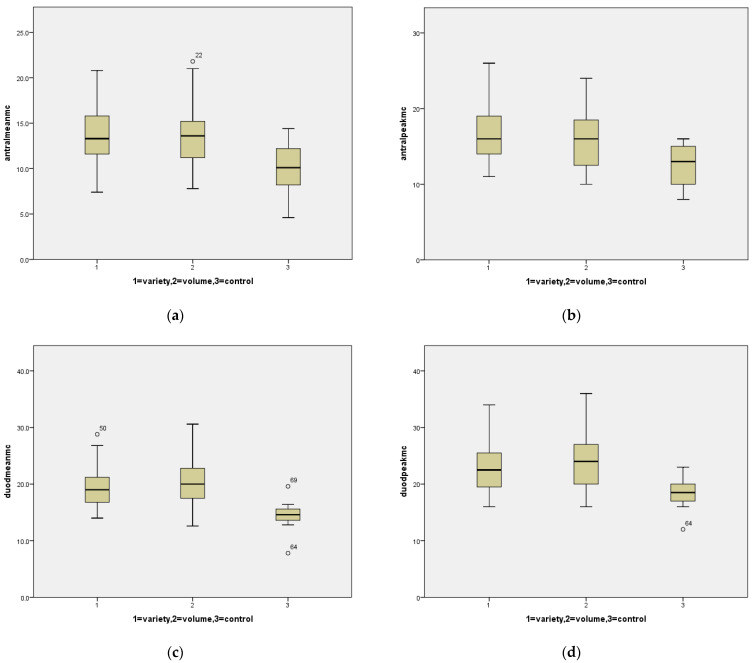
Box plots for mast cells comparing the three groups: (**a**) mean mast cell counts in the antrum for variety limited, volume limited and controls; (**b**) peak mast cell counts in the antrum for variety limited, volume limited and controls; (**c**) mean mast cell counts in the duodenum for variety limited, volume limited and controls; (**d**) peak mast cell counts in the duodenum for variety limited, volume limited and controls. Circles represent outliers.

Six hundred and sixty-three patients were seen for evaluation in the Multidisciplinary Feeding Clinic between 1 October 2014 and 31 December 2019. Three hundred and thirty six of these had undergone an EGD. Of these, we identified 30 patients with volume limitation without complaint of variety limitation and 30 patients with variety limitation without volume limitation. The mean ages for Volume vs. Variety were 4.2 years ± 3.1 vs. 4.2 years ± 3.0; *p* = 0.948. Mean and peak eosinophil and mast cell counts are listed in Table 1, with a comparison of each of the three groups in Figure 1. 

Utilizing a cut-off value of 10 eosinophils/hpf, increased gastric eosinophils were seen in 10.7% of variety-limited patients and 6.3% of volume-limited patients. Utilizing a cut-off value of 20 eosinophils/hpf, increased duodenal eosinophils were seen 46.4% of variety-limited patients and 37.5% of volume-limited patients. If this value was increased to 30/hpf, increased duodenal eosinophils were seen in 14.3% of variety-limited patients and 12.5% of volume-limited patients.

For mast cells, frequencies were assessed utilizing cut-off values determined by the mean + [2 × standard deviation] in the control group which yielded a cut-off value of 19.9 in the antrum and 24.0 in the duodenum. Utilizing these cut-off values, antral mast cell density was increased in 32.1% of variety-limited patients, 37.5% of volume-limited patients, and in no controls; duodenal mast cell density was increased in 32.1% of variety-limited patients, 40.6% of volume-limited patients, and in no controls.

## 4. Discussion

It has been established that mast cells play a role in multiple pediatric gastrointestinal disorders including eosinophilic esophagitis, functional dyspepsia, and irritable bowel syndrome [15]. This was the first study to evaluate possible mast cell involvement in patients with feeding difficulties. In both variety- and volume-limited patients, antral and duodenal mast cell densities were increased and did not differ between the two groups. A role for mast cells in feeding difficulties is biologically plausible and possibly related to increased gastric sensitivity. Mast cell infiltration and activation has been shown to increase visceral sensitivity in both animal models and humans [16,17,18,19]. Mast cell infiltration and activation could account for hypersensitivity to stretch and possibly also for chemical sensitivity as has been demonstrated in adults with functional dyspepsia with enteral lipid infusion [20,21]. In children with functional dyspepsia, antral mast cell density is increased in those with the postprandial distress syndrome subtype, which is marked by the presence of early satiety and postprandial bloating, sensations which may limit intake [22]. In addition, increased mast cells have been associated with delayed gastric emptying in the early postprandial phase in patients with functional dyspepsia [9]. There has been one small study evaluating gastric emptying in infants with both cow’s milk protein allergy and gastroesophageal reflux, which found a significant difference in delayed emptying in children with cow’s milk protein allergy, a condition associated with mast cell activation, when compared to gastroesophageal reflux [23]. It is possible there was a similar mechanism contributing to the volume and variety limitations in this subset of patients with feeding difficulties. Furthermore, mast cells have been associated with nausea in patients with functional GI disorders [8], which is a symptom that would not be possible to obtain from young children but could possibly manifest as feeding difficulties. These associations need further investigation to understand the mechanism behind the presence of mast cells and development of feeding difficulties in children, which will allow us to provide more targeted therapies.

Due to the absence of well-defined, agreed-upon criteria for the diagnosis of pediatric eosinophilic gastrointestinal disorders, we chose to report absolute counts of eosinophils, versus reporting the presence or absence of eosinophilic gastrointestinal disorders. Although overall eosinophil densities did not differ from controls, a substantial portion of both variety- and volume-limited patients had elevated duodenal eosinophils. Mukadda and colleagues evaluated the prevalence of feeding disorders in patients with eosinophilic GI disorders and demonstrated an increased prevalence, including 90.9% of those with low variety of intake and 81.8% with low volume of intake [14]. In their population of patients with eosinophilic gastrointestinal diseases, they found maladaptive feeding behaviors to be the primary type of feeding difficulty, followed by gagging and vomiting. It is possible this is analogous to the symptoms observed in children with functional dyspepsia and eosinophilia. 

This study was limited by its retrospective design and inability to control for comorbidities and medical complexity of the patient, including history of atopy. There was also the possibility of bias in the selection of patients for endoscopy. An additional limitation includes variability with regard to how symptoms are reported by parents. Lastly, our sample size of 30 patients with volume and variety limitation each may not be reflective of the entire pediatric population with these same feeding difficulties. 

This study was targeted at understanding if there was a significant increase in eosinophils or mast cells in patients with volume and variety limitations among children with feeding difficulties. Our study demonstrates the presence of increased mast cells, but future studies are needed to further understand the etiology of the increased mast cells and determine if treatments targeting eosinophils, mast cells, or their mediators are beneficial to patients.

## 5. Conclusions

In conclusion, we have demonstrated an increase in antral mast cell density in children with both volume-limited and variety-limited feeding difficulties. These findings suggest a potential role for mast cells in the generation of feeding difficulties and potentially a viable therapeutic target. Future studies should address the efficacy of treatments targeting mast cells in this patient group.

## Figures and Tables

**Table 1:** Means (± SD) for cell counts across groups.

	Variety Limited	Volume Limited	Controls	*p* Values
Antral mean eosinophils	4.8 ± 4.4	4.8 ± 5.7	3.1 ± 1.6	0.485
Antral peak eosinophils	7.0 ± 6.3	7.4 ± 8.7	4.9 ± 2.1	0.745
Duodenal mean eosinophils	16.3 ± 8.3	15.8 ± 5.6	13.2 ± 4.7	0.722
Duodenal peak eosinophils	21.0 ± 11.9	20.6 ± 8.7	17.8 ± 6.2	0.940
Antral mean mast cells	13.8 ± 3.4	13.7 ± 3.5	10.0 ± 3.0	0.015
Antral peak mast cells	16.6 ± 3.9	16.4 ± 4.2	12.5 ± 2.7	0.012
Duodenal mean mast cells	19.4 ± 3.6	20.8 ± 4.4	14.4 ± 3.0	<0.001
Duodenal peak mast cells	22.7± 4.4	24.1 ± 5.2	18.2 ± 2.9	0.003

## Data Availability

The dataset is available from the corresponding author on request.
